# Nocardia rubra cell-wall skeleton activates an immune response in cervical tissue *via* stimulating FPR3 to enhance dendritic cell-mediated Th1 differentiation

**DOI:** 10.3389/fimmu.2023.1117545

**Published:** 2023-03-02

**Authors:** Qianyu Guo, Wei Chen, Junyi Sun, Chunfang Zhao, Xue Bai, Yanan Zhang, Ke Liu, Lei Zhang, Suxia Shao

**Affiliations:** ^1^ Department of Histology and Embryology, Hebei Medical University, Shijiazhuang, China; ^2^ Department of Gynecology, The Fourth Hospital of Hebei Medical University, Shijiazhuang, China

**Keywords:** human papillomavirus (HPV), cervical intraepithelial neoplasia (CIN), immunotherapy, dendritic cells (DCs), Th1 cell polarization, formyl peptide receptor 3 (FPR3)

## Abstract

*Nocardia rubra* cell wall skeleton (Nr-CWS) has proven to be a successful medicine for therapy of cervical human papillomavirus infection. The mechanism of action of Nr-CWS is unclear but may involve a stimulatory effect on the host immune system. We previously found that CD4^+^ T cells were increased in cervical tissue after Nr-CWS treatment. Microarray data from these cervical tissues revealed the significant upregulation of formylated peptide receptor 3 (FPR3). This study aimed to explore the role of Nr-CWS in immunomodulatory based on these findings. Examination of CD4^+^ T cell subsets in cervical tissue from patients who received Nr-CWS revealed substantial increases in Th1 cytokines and transcription factors. The regulatory effects of Nr-CWS on the function and phenotype of dendritic cells (DCs) were assessed in comparison with the traditional DC maturation inducer lipopolysaccharide (LPS). Similar to LPS, Nr-CWS potently induced DC maturation and interleukin-12 (IL-12) secretion. Differentiation of T cells induced by Nr-CWS stimulated DCs was assessed using the mixed lymphocyte reaction assay. Significant differentiation towards Th1 was evident. Finally, FPR3 expression in DCs in response to Nr-CWS and LPS was measured. Nr-CWS potently upregulated FPR3 expression, while the LPS did not. Silencing FPR3 in DCs reduced Nr-CWS-induced IL-12 production and Th1 cell polarization in co-cultured T cells. The collective findings indicate that Nr-CWS may target FPR3 on the surface of DC cells and activate a Th1-type immune response. The findings clarify the basis of the antiviral immune effects of Nr-CWS on human papillomavirus.

## 1 Introduction

Cervical cancer is the leading cause of death for women in developing countries (1). Persistent infection with high-risk human papillomavirus (HR-HPV) is responsible for almost all cervical cancers and precancerous lesions (2). The availability of a vaccine and cytological screenings have gradually decreased the incidence of cervical cancer. However, clearance of long-term persistent HPV infection is still the central dilemma in the integrative treatment of precancerous lesions.

CD4^+^ T cells comprise a group of cell subtypes that include helper T cells (Th1, Th2, Th17) and regulatory T cells (Treg). These cells are essential in mediating HPV infection (3, 4). Th1-type inflammatory responses characterized by secretion of interferon-gamma (IFN-γ) and interleukin-2 (IL-2) are necessary for HPV clearance (5). In contrast, Th2-type inflammatory reactions are responsible for persistent HPV infections and cervical dysplastic development *via* immune suppression, which is characterized by the production of IL-4 and IL-10 (6). Th17 cells secrete IL-17, which has been positively correlated with the progression of cervical lesions (7). Tregs recruited by HPV are immunosuppressive. In contrast to regressing epithelial lesions, infiltration of Treg cells (Foxp3 positive cells) increases in cervical tissues as the cervical lesion progresses (8–10).

*Nocardia rubra* cell wall skeleton (Nr-CWS) has been used for the treatment of squamous intraepithelial lesions and is effective in both regressing lesions and clearing HPV infections (11). Nr-CWS might have immuno-modulating effects by directly activating the CD4^+^ and CD8^+^ T cell immunological responses (12, 13). Our previous study found that local treatment with Nr-CWS increased the number of CD4^+^ T cells in the cervical tissue (14). However, whether Th1-type immune responses are enhanced locally remains unclear.

Antigen-presenting cells bridge innate and adaptive immune systems. Dendritic cells (DCs), which control the destiny of naïve CD4^+^ T cells, are vital for inducing antiviral immune responses (15). Antigenic stimulation leads to the maturation of DCs, which is characterized by increased expressions of HLA-DR, CD80, CD83, and CD86 (16–18). Recently, it has been proposed that Nr-CWS facilitates the proliferation and viability of DCs (19). Zhang et al. reported that Nr-CWS can promote the maturation of DCs (20). Based on these findings, we explored the response of T cells to DCs stimulated by Nr-CWS.

Our unpublished microarray data revealed the significant upregulation of formylated peptide receptor 3 (FPR3) in cervical tissue from patients with effective HPV clearance by Nr-CWS. FPR3 is a member of the FPR family. The protein performs critical functions in the immune system’s initial identification of infection by detecting pathogen-associated molecular patterns that signify the presence of bacteria (21). FPR3 is only expressed in monocytes and DCs, and few ligands have been identified (22). As one of the most recently identified members, the overall function of FPR3 remains to be determined. FPR3 is reportedly a negative regulator of LPS-induced DC maturation and T cell activation (23), and participates in sensitizing lipoprotein-mediated Th2 cell differentiation (24).

In the present study, the effect of Nr-CWS on DC-induced T cell differentiation was explored *in vivo* and *in vitro*. The role of FPR3 in this process was assessed.

## 2 Materials and methods

### 2.1 Patients

A total of 72 patients ranging in age from 25 to 64 years (mean 41.3 ± 7.3 years) with HR-HPV infection and histological abnormalities were recruited from the Department of Gynecology of The Fourth Hospital of Hebei Medical University, Hebei, China, from 2019 to 2021. Pregnant patients and those with severe heart, lung, liver, and kidney diseases, other neoplastic diseases, immunocompromised state, and those receiving any anti-inflammatory or immunosuppressive treatment were excluded. Clinical and demographic characteristics of patients are described in **Table 1**. As previously described (14), all patients received adequate treatment with Nr-CWS. Briefly, the treatment comprised topical application of 120 μg Nr-CWS to the cervix on alternate days during a menstrual cycle for a total of 10 times. All applications were performed by a specialist physician. Cervical tissue samples were collected before (Case group) and 30 days (Nr-CWS 30D group) or 90 days (Nr-CWS 90D group) following topical administration of Nr-CWS.

**Table 1:** Clinical and demographic characteristics of patients.

	Normal group	Nr-CWS groups		
		Case	Nr-CWS 30D	Nr-CWS 90D
Number	8	72	31	41
Age range (years)	28-41	25-64	–	–
Mean age (years)	35.4 ± 3.1	41.3 ± 7.3	–	–
HR-HPV infection type				
Single infection	–	47	21	26
Multiple infection	–	25	10	15
Pathological features				
CIN I	–	65	29	36
CIN II	–	7	2	5

Normal cervical samples were collected from HPV-negative patients (Normal group, n=8, age range 28-41 years, mean 35.4 ± 3.1 years) who had a complete hysterectomy for uterine fibroids. No medication was administered before the surgery. All cases were histologically proven.

This study was approved by the Ethics Committees of The Fourth Hospital of Hebei Medical University (2019MEC097). Written informed consents were obtained from all patients. The study complied with the Declaration of Helsinki protocols.

### 2.2 Immunohistochemical staining (IHC)

Tissues were fixed in 4% buffered formalin, descaled by EDTA, embedded in paraffin, and sectioned. Slices 5μm in thickness were incubated overnight with the following primary antibodies (1:400 dilution for each): IFN-γ (Cat#: DF-6045; Affinity Biosciences, USA); IL-4 (Cat#: AF-5142; Affinity Biosciences),IL-17a (Cat#: ab79056; Abcam, USA), Foxp3 (Cat#: ab215206; Abcam) and transforming growth factor-beta (TGF-β, Cat#: CY2179; Abways Technology, USA). Polymeric secondary antibodies coupled to horseradish peroxidase (ZsBio, China) and 3, 3’-diaminobenzidine (DAB) were used for visualization of antibody binding. Immunohistochemical analysis was performed as described previously (14).

### 2.3 RNA isolation and PCR

Tissue samples for PCR were snap-frozen in liquid nitrogen after isolation and were stored at -80°C until use. Total RNA isolated from tissues using RNAprep pure Micro Kit (Cat#: DP420; Tiangen, China) was reverse-transcribed to cDNA using PrimeScript™ RT reagent Kit with a genomic DNA Eraser (Cat#: RR047A; TaKaRa Bio, Japan). Quantitative PCR was performed by QuantStudio3 (Applied Biosystems, USA) with TB Green^®^ Premix Ex Taq™ II (Cat#: RR820B; TaKaRa Bio) according to the manufacturer’s instructions. β-actin was used as the housekeeping gene. Gene expression was calculated by the 2^−ΔΔCT^ method. Primer sequences are listed in **Table 2**.

**Table 2:** Forward and reverse primer sequences for transcription factors.

Primer name	Oligo sequence (5′ to 3′)
T-bet Forward	CCCCAGTACCCTCCCAAGAT
T-bet Reverse	TTCGCCCAGTCCTGAATCAC
GATA3 Forward	AAGGCAGGGAGTGTGTGAAC
GATA3 Reverse	AGCCTTCGCTTGGGCTTAAT
RORγt Forward	GCTGATGGGAACGTGGACTA
RORγt Reverse	CCCACGGACACCAGTATCTT
Foxp3 Forward	ACTGGGGTCTTCTCCCTCAA
Foxp3 Reverse	GACACCATTTGCCAGCAGTG
β-actin Forward	CGCCACCAGTTCGCCATGGA
β-actin Reverse	TACAGCCCGGGGAGCATCGT

### 2.4 Cell preparations

#### 2.4.1 DCs

DCs were obtained using a slight modification of a previously described method (25). Leukocyte reduction system (LRS) chambers from anonymous blood donors were obtained from the Hebei Provincial Blood Center (Shijiazhuang, China) according to the guidelines of the local blood bank. This acquisition was approved by Hebei Medical University. Human monocyte-derived DCs were derived from peripheral blood mononuclear cells (PBMCs) obtained from the LRS chambers. The DCs were cultured with 30 ng/ml recombinant human granulocyte-macrophage colony-stimulating factor (rhGM-CSF) (Cat#: 30003; Peprotech, USA) and 30 ng/ml rhIL-4 (Cat#: 20004; Peprotech) for 5 days. DCs were divided into three groups: cells treated with phosphate-buffered saline (immature DC [iDC] group), cells treated with 30 μg/ml Nr-CWS (Liaoning Greatest Bio-Pharmaceutical Co. Ltd., China) (Nr-CWS group), and cells treated with 100 ng/ml lipopolysaccharide (LPS, Cat#: L4516, Sigma-Aldrich, USA) (LPS group) for 2 days. The medium was collected for cytokine analysis and the cells were analyzed by flow cytometry.

#### 2.4.2 T cells

Naïve CD4^+^ T cells were isolated from PBMCs using the Human CD4^+^ Naïve T Cell Isolation Kit (Cat#: 480042; BioLegend, USA).

#### 2.4.3 Co‐culture experiments

The mixed lymphocyte reaction (MLR) of the co-culture of DCs and T cells was performed to examine the ability of DCs to stimulate T cells. DCs (1×10^5^ cells per well) and allogeneic naïve CD4^+^ T cells (1×10^6^ cells per well) were co-cultured for 3 days, and the medium was collected for cytokine analysis. HeLa cells were plated onto 3 μm Falcon Transwell^®^ membranes (Corning, USA) at a density of 5×10^4^ cells per well. When cell growth reached 80% confluency, DC and T cells were co-cultured in the lower chamber of the culture plates for 2 days.

### 2.5 Flow cytometry

After 2 days treatment of Nr-CWS or LPS, DCs were washed, centrifuged, and resuspended in 200 µl PBS containing a 1:40 dilution (5 μg/ml) of antibodies to CD83 (Cat#: 305327; BioLegend), CD86 (Cat#: 305405; BioLegend), HLA-DR (Cat#:307603; BioLegend), and FPR3 (Cat#: ab172908; Abcam). After incubation at 4 °C for at least 1 h, cells were washed and resuspended in PBS buffer. Flow cytometry analysis was performed using the BD Accuri C6 flow cytometer instrument (BD Biosciences, USA). Data were analyzed by FlowJo v10 software (FlowJo, USA).

### 2.6 Scanning electron microscopy (SEM)

DCs and MLR co-cultures were prepared for SEM. Cells were fixed in 2.5% glutaraldehyde buffer for 30 min and then dehydrated through an alcohol gradient. After being completely air-dried, the samples were coated with gold particles and observed with a model 8100 microscope (Regulus, Japan).

### 2.7 Small interfering RNA (siRNA) transfection

The siRNAs were purchased from GenePharma (China). The sequences were: si-FPR3, 5’-GCCAUCCUACCAUUCCGAATT-3’ and si-scrambled: 5’-UUCUCCGAACGUGUCACGUTT-3’. On day 1 and 3 of the DC culture, siRNAs were transfected using Transfection Reagent Ultra Fection 3.0 (Cat#: FXP135; 4A Biotech, China) at a final concentration of 20 nM according to the manufacturer’s instructions. On day 5, DCs were harvested and used for further experiments.

### 2.8 Enzyme-linked immunosorbent assay (ELISA)

Medium collected from cell culture was used for ELISA analyses of cytokines (Abclonal, China) according to the manufacturer’s instructions. The cell supernatants were collected on day 7 of DC culture and analyzed for IL-12p70 (Cat#: RK00014) and IL-10 (Cat#: RK00012). Cell supernatants of co-cultures of DC and naïve CD4^+^ T cells were collected after 2 days of MLR and analyzed for IL-2 (Cat#: RK00002), IFN-γ (Cat#: RK00015), IL-4 (Cat#: RK00003), TGF-β (Cat#: RK00055) and IL-17a (Cat#: RK00397).

### 2.9 CCK-8 assay of cell proliferation

The optimal concentration of Nr-CWS was analyzed by Cell Counting Kit -8 (CCK-8) assay (Cat#: C0038; Beyotime, China). On day 5 of induction culture of human monocyte-derived DCs, different concentrations of Nr-CWS (3.75 to 60 µg/ml) were added. After cultured for 2 days, cells were harvested and analyzed using the CCK-8 assay. The proliferation of HeLa cells was detected with CCK-8 after 2 days of co-culture with MLR cultures in Transwell units.

### 2.10 Statistical analyses

Data are shown as mean ± SD. Statistical analyses were performed using SPSS software version 25.0 (SPSS Inc., USA). Statistical significance was assessed using the one-way analysis of variance (ANOVA) or independent samples T-test. Significance was evident as *P*<0.05.

## 3 Results

### 3.1 Nr-CWS promotes Th1 differentiation in cervical tissue

**Figure 1:**
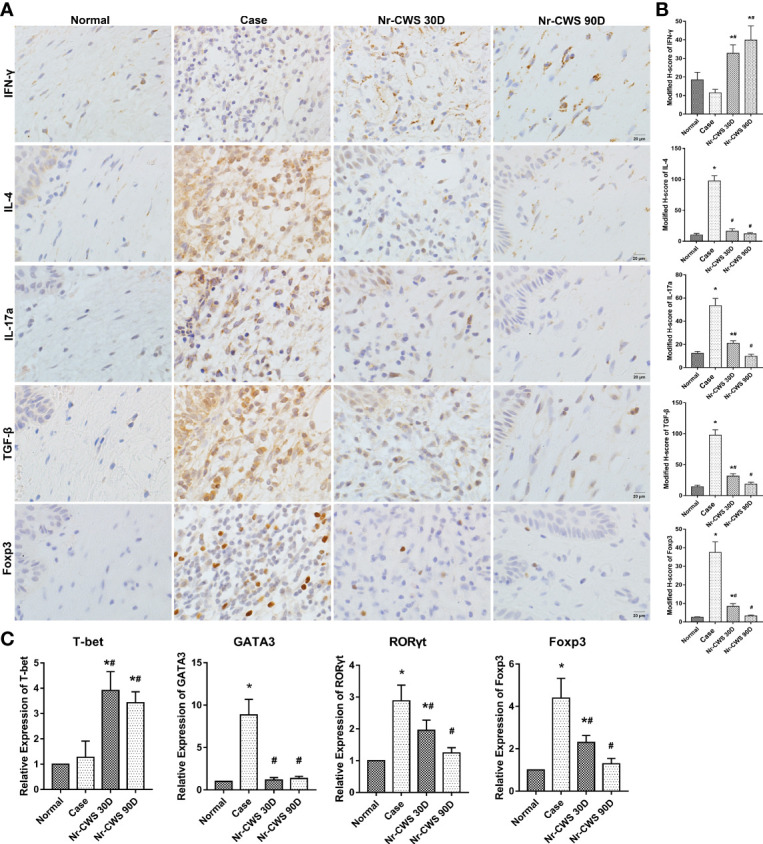
Effects of Nr-CWS on CD4^+^ T cells in cervical tissue of cervical dysplasia patients. **(A)** Representative immuno-histochemistry images of CD4^+^ T cell subsets in cervical tissue. Cervical samples were collected from non-HPV patients (Normal), HPV+ patients before Nr-CWS treatment (Case), and 30 days (Nr-CWS 30D) and 90 days (Nr-CWS 90D) after Nr-CWS. IL-4, IFN-γ, IL-17, TGF-β, and Foxp3 expression were checked by immuno-histological staining. Scale bars = 20 μm. **(B)** Modified H-scores for the expression of Foxp3, IL-4, IFN-γ, IL-17a, TGF-β, and IFN-γ in cervical tissue. **(C)** Relative mRNA expression of T-bet, GATA3, RORγt, and Foxp3 in cervical tissue samples was analyzed by qPCR (n=35). *P < 0.01, vs. Normal group; ^#^ *P* < 0.01, vs. Case group.

Compared to the normal group, patients infected with HPV displayed higher expressions of IL-4, IL-17, TGF-β, and Foxp3 in cervical tissue (all *P*<0.05). These increases were markedly reduced by the 30-day Nr-CWS treatment, with the reductions being even more pronounced after 90 days (**Figures 1A, B**, *P*<0.05). IFN-γ expression was found lower in both the normal and case groups. The expression was dramatically increased after 30 and 90 days of Nr-CWS therapy (**Figures 1A, B**, *P*<0.05). The findings indicate that Nr-CWS treatment may improve local immune response by enhancing the Th1 type response. Gene expressions of major transcription factors involved in T cell development in cervical tissue were measured. T-bet, a transcription factor that represents Th1 cells, was significantly increased in cervical tissue following Nr-CWS therapy. In contrast, GATA3 expression by Th2 cells, RORγt expression by Th17 cells, and Foxp3 expression by Treg cells were reduced following Nr-CWS therapy (**Figure 1C**, *P*<0.05).

### 3.2 Nr-CWS promotes maturation of DCs

**Figure 2:**
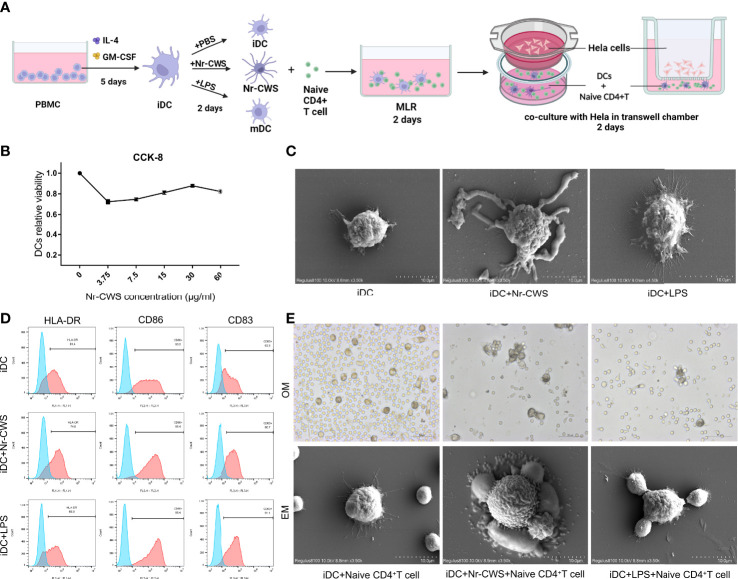
Nr-CWS promote maturation of DCs. **(A)** Schematic diagram of the *in vitro* experimental process. **(B)** DC activity was measured by CCK-8 after 48 h of stimulation with different doses of Nr-CWS. Results are representative of five independent experiments. **(C)** HLA-DR, CD83, and CD86 expression in DCs was measured by flow cytometry. **(D)** Scanning electron microscopy (SEM) observation of morphological alterations of DC cells. **(E)** Optical microscope (OM) and SEM observations of the stimulated DCs attraction to naïve T cells.

The cell culture procedure is shown in **Figure 2A**. CCK8 assay results showed that the optimal concentration of Nr-CWS was 30 μg/ml (**Figure 2B**). This concentration was used in further experiments. To evaluate the alterations in DCs after Nr-CWS stimulation, we examined the morphology of DCs, evaluated the surface markers of DCs maturation, and co-cultured DCs with naïve CD4^+^ T cells to examine their capacity to attract T cells. Unstimulated iDCs and LPS-stimulated mature DCs (mDCs) were used as controls. Compared with iDCs, Nr-CWS treatment led to significantly increased expression of maturation markers CD86, CD83 and HLA-DR in DCs, indicating that Nr-CWS promotes maturation of DCs (**Figure 2C**). The maturation of DCs was accompanied by increases in size and protrusion. DCs exposed to Nr-CWS had a larger volume, with unique pseudopod-like cell protrusions and uneven surface. LPS-induced mDCs featured lengthy, burr-like cell protrusions (**Figure 2D**). In addition, Nr-CWS treatment induced DCs to attract naïve T cells. In co-cultures of DCs and T cells, iDCs included dispersed DCs that failed to recruit T cells. Naïve T cells that were connected *via* cell protrusions gathered around Nr-CWS treated DCs. The appearance was comparable to the effects of LPS (**Figure 2E**).

### 3.3 DCs treated with Nr-CWS induce Th1 cell differentiation and impede proliferation of HeLa cells

**Figure 3:**
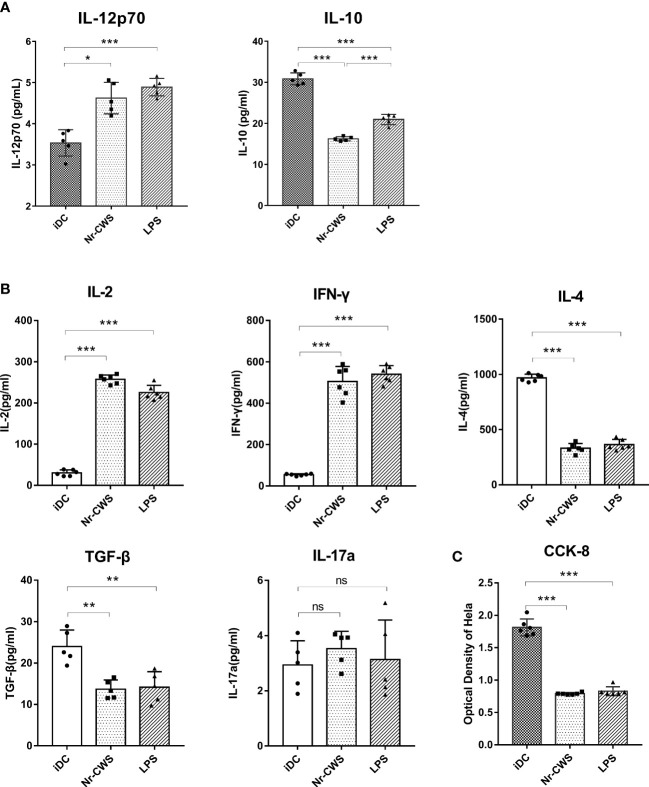
Nr-CWS induced Th1-type cell immune responses through DCs. **(A)** ELISA data of IL-12p70 and IL-10 cytokines in the medium. **(B)** ELISA data of IL-2, IFN-γ, IL-4, TGF-β, and IL-17a cytokines in the medium of DC-T cell co-cultures. **(C)** CCK-8 data for HeLa cell proliferation (n=5). *P < 0.05, **P < 0.001, and ***P < 0.0001, vs iDC. NS, No significance. *P < 0.05, **P < 0.01, and ***P < 0.001.

IL-12p70 is necessary for Th1 activation (26), whereas IL-10 operates as a Th2-type cytokine. In the present study, compared to iDCs, Nr-CWS and LPS increased IL-12p70, while reducing IL-10 production in DCs (**Figure 3A**, all *P*<0.05). Allogeneic naïve CD4^+^ T cells were added to DCs in MLR co-cultures. The culture medium was collected and secretion from T cells was determined by ELISA. Compared to iDCs, Nr-CWS treatment led to significantly increased IL-2 and IFN-γ levels, while reducing IL-4 and TGF-β levels in the medium (**Figure 3B**, *P*<0.05). In contrast, IL-17a showed no significant differences across groups. Nr-CWS-stimulated DCs displayed significantly induced Th1 differentiation. Hela cells were treated with DC-T co-culture medium in Transwell chambers, and proliferation was measured by the CCK-8 assay. Compared to the iDC group, proliferation of HeLa cells in the Nr-CWS group was significantly inhibited (**Figure 3C**, *P*<0.05), indicating that the immune response activated by Nr-CWS may inhibit the proliferation of HeLa cells.

### 3.4 Nr-CWS upregulates FPR3 expression in cervical tissues and DCs

**Figure 4:**
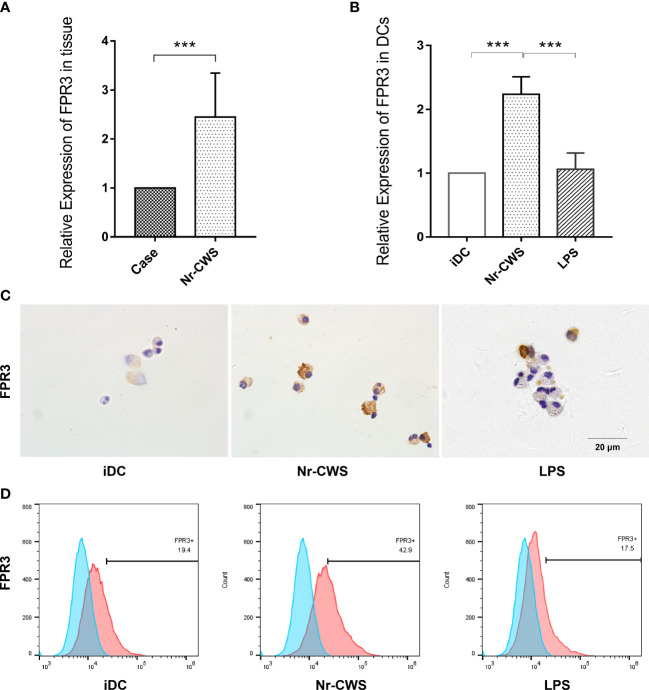
Nr-CWS upregulates FPR3 expression in cervical tissue and DCs. **(A)** FPR3 mRNA expression in cervical tissue (n=5). **(B)** FPR3 mRNA expression in DCs (n=3). **(C)** Immunohistochemical staining of FPR3 in DCs. **(D)** Flow cytometry detection of FPR3 expression in DCs. ***P< 0.001.

Our previous unpublished microarray data from the cervical tissue of patients after Nr-CWS treatment revealed a significant increase in FPR3, which was uniquely expressed in DCs. In the present study, FPR3 expression in cervical tissue was significantly higher as determined by PCR (**Figure 4A**, *P*<0.05). The finding indicates d higher FPR3 expression in Nr-CWS-treated patients. Similar results were obtained *in vitro* studies; compared with iDCs, Nr-CWS led to significantly increased FPR3 expression in DCs at both mRNA and protein levels (**Figures 4B–D**, *P*<0.05). However, similar alterations were not observed in LPS-induced mDCs (**Figures 4B–D**, *P*>0.05). Therefore, Nr-CWS might achieve its effects through the upregulation of FPR3 expression.

### 3.5 FPR3 participates in Th1 differentiation induced by Nr-CWS

**Figure 5:**
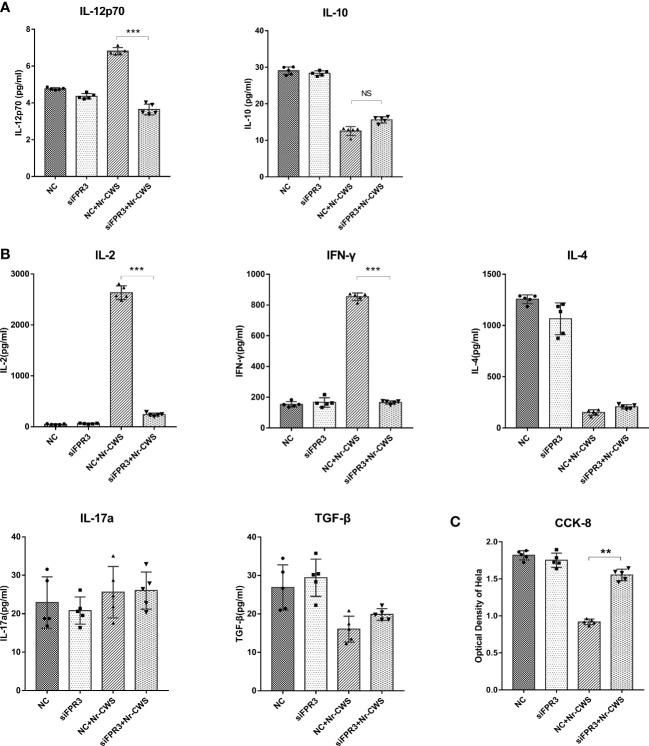
Effects of Nr-CWS involve FPR3 activation. **(A)** ELISA data of IL-12p70 and IL-10 cytokines in the medium of DCs. **(B)** ELISA data of IL-2, IFN-γ, IL-4, TGF-β, and IL-17a cytokines in the medium of DC-T cell co-cultures. **(C)** CCK-8 data for HeLa cell proliferation in medium of co-cultured with DCs and T cells following stimulation of DCs with Nr-CWS (n=5). ***P < 0.01, vs iDC. NC, Negative control. NS, No significance. **P < 0.01, ***P < 0.001.

Compared to the NC+Nr-CWS group, silencing of FPR3 led to significantly reduced cellular IL-12 production in DCs of siFPR3+Nr-CWS group (**Figure 5A**, *P*<0.05), indicating that the effect of Nr-CWS on DC induction and T cell immunity activation was largely due to FPR3 activation. Compared to the NC+Nr-CWS group, the effect of Nr-CWS on the production of IFN-γ and IL-2 was significantly attenuated by FPR3 knockdown (**Figure 5B**, *P*<0.05). However, silencing of FPR3 had no effects on the levels of IL-4, IL-17a, and TGF-β in Nr-CWS treated cells, indicating that FPR3 is the key factor involved in Th1 differentiation induced by Nr-CWS in DCs (**Figure 5B**). Likewise, the inhibition of HeLa cell proliferation by Nr-CWS was also impaired in cells in which FPR3 had been knocked down (**Figure 5C**, *P*<0.05). Therefore, interfering with FPR3 activation would prevent Nr-CW-treated DCs from polarizing naïve CD4^+^ T cells towards Th1, suggesting that Nr-CWS induces Th1 production *via* FPR3.

## 4 Discussion

HPV can escape from host immune surveillance by creating an immunosuppressive microenvironment locally. Whether HPV is cleared or persists depends on the interaction of immune escape mechanisms and host immune responses (27). The eradication of HPV infection is the most important treatment for preventing early lesions from developing into cervical cancer.

The clinical effects of Nr-CWS on HPV infection and cervical lesions may be due to its immune activation of T cells (14). The present study is the first to explore the effect of Nr-CWS on CD4^+^ T cell subsets in cervical tissue of HPV-infected patients and found an enhanced Th1 immune response. Nr-CWS may stimulate DC maturation and induce naïve CD4^+^ T cell polarization towards Th1 cells.

Th1 cells that develop from CD4^+^ T cells are crucial for intracellular killing immune responses against HPV (28, 29). Studies have demonstrated fewer Th1 responses, but enhanced Th2 responses, to HPV-infected cervical tissue (30, 31). Th17 is increased in cervical dysplasia and may contribute to immunosuppression (32). The presence of Treg cells is correlated with a poor prognosis in tumors and contributes to HPV evasion of host immune surveillance (10, 33, 34). In the present study, Nr-CWS therapy dramatically increased the production of the Th1 cytokine IFN-γ, while decreasing the expression of Th2, Th17, and Treg-related cytokines in cervical tissue, suggesting Nr-CWS enhances the immune microenvironment against viral infection in cervical tissue.

DCs are the initiating trigger for T cell immunity. Migration of iDCs is pronounced, while mature DCs activate naïve T cells and drive the development of these cells into effector cells (16, 35). Maturation of DCs can be promoted by bacterial components, inflammatory cytokines, or antigen-antibody complexes (36). Nr-CWS is a bacterial-derived extract that activates DCs *in vitro* (20). In the present study, Nr-CWS enhanced HLA-DR, CD86, and CD83 expression in DCs, increased IL-12 production, and reduced IL-10 production. IL-10 and -12 are markers of Th1 differentiation. In addition, we found that DCs activated by Nr-CWS could induce Th1 differentiation, which was characterized by IFN-γ secretion. IFN-γ reportedly causes programmed death in HeLa cells (37, 38). Co-culture experiments with HeLa cells have also shown growth inhibition due to Nr-CWS.

FPR3 is continuously expressed during the maturation of bone marrow-derived DCs and may regulate the DCs of transport-T cell immunostimulatory phase by targeting unknown ligands (39). In the present study, Nr-CWS stimulated FPR3 expression in DCs, and FPR3 participated in Nr-CWS-induced Th1 differentiation. However, some ligands can promote FPR3 activation and prevent LPS-induced DC maturation and IL-12 secretion (23). Another study reported that sensitizing lipid transport proteins bind to FPR3 in DCs and inhibit T cells by releasing IL-12 and facilitating the generation of IL-10 (24). The activation of FPR3 results in a Th2-type immune response. Therefore, Nr-CWS-stimulated FPR3 activation in the present study led to a different type of immunity from what has been previously reported. Activation of FPR3 on DCs by different ligands showed different effects on the direction of T cell differentiation. Thus, FPR3 processing in DCs for antigen presentation and guidance of T cell development orientation may depend on ligand signaling. Peptide elements of Nr-CWS might be new FPR3 ligands that activate IFN-dominated antiviral immune responses. Further studies are necessary to explore this suggestion.

In summary, Nr-CWS induces Th1 immune responses in the cervical tissue of HPV-infected patients. Nr-CWS may induce DC cell maturation and direct Th1 cell differentiation by stimulating FPR3 expression on DCs. This study provides novel evidence of the influence of Nr-CWS on immunotherapy.

## Data availability statement

The original contributions presented in the study are included in the article/supplementary material. Further inquiries can be directed to the corresponding authors.

## Ethics statement

The studies involving human participants were reviewed and approved by the Ethics Committees of the 4th hospital of Hebei Medical University. The patients/participants provided their written informed consent to participate in this study.

## Author contributions

LZ and SS contributed to the conception and design of the study. QG designed and performed experiments, analyzed the samples and contributed to the manuscript preparation. WC contributed to the design of the experiments and study supervision. JS collected LRS chambers, performed experiments and analyzed the data. CZ performed experiments and contributed to the project administration. XB was responsible for clinical patient recruitment and treatment. YZ and KL collected samples from the patients and performed experiments. All authors contributed to the article and approved the submitted version.
